# Comparing the Prognostic Accuracy for All-Cause Mortality of Frailty Instruments: A Multicentre 1-Year Follow-Up in Hospitalized Older Patients

**DOI:** 10.1371/journal.pone.0029090

**Published:** 2012-01-11

**Authors:** Alberto Pilotto, Franco Rengo, Niccolò Marchionni, Daniele Sancarlo, Andrea Fontana, Francesco Panza, Luigi Ferrucci, on behalf of the FIRI-SIGG Study Group

**Affiliations:** 1 Geriatrics Unit, Azienda ULSS 16 Padova, S. Antonio Hospital, Padova, Italy; 2 Gerontology-Geriatrics Research Laboratory, Institute of Care and Scientific Research “Casa Sollievo della Sofferenza”, San Giovanni Rotondo, Foggia, Italy; 3 Chair of Geriatrics, Federico II University, Napoli, Italy; 4 Salvatore Maugeri Foundation, Institute of Care and Scientific Research, Benevento, Italy; 5 Geriatric Department, University of Florence, Florence, Italy; 6 Unit of Biostatistics, Institute of Care and Scientific Research “Casa Sollievo della Sofferenza”, Foggia, Italy; 7 National Institute on Aging, Longitudinal Studies Section, Harbor Hospital Center, Baltimore, Maryland, United States of America; University of Valencia, Spain

## Abstract

**Background:**

Frailty is a dynamic age-related condition of increased vulnerability characterized by declines across multiple physiologic systems and associated with an increased risk of death. We compared the predictive accuracy for one-month and one-year all-cause mortality of four frailty instruments in a large population of hospitalized older patients in a prospective multicentre cohort study.

**Methods and Findings:**

On 2033 hospitalized patients aged ≥65 years from twenty Italian geriatric units, we calculated the frailty indexes derived from the Study of Osteoporotic Fractures (FI-SOF), based on the cumulative deficits model (FI-CD), based on a comprehensive geriatric assessment (FI-CGA), and the Multidimensional Prognostic Index (MPI). The overall mortality rates were 8.6% after one-month and 24.9% after one-year follow-up. All frailty instruments were significantly associated with one-month and one-year all-cause mortality. The areas under the receiver operating characteristic (ROC) curves estimated from age- and sex-adjusted logistic regression models, accounting for clustering due to centre effect, showed that the MPI had a significant higher discriminatory accuracy than FI-SOF, FI-CD, and FI-CGA after one month (areas under the ROC curves: FI-SOF = 0.685 vs. FI-CD = 0.738 vs. FI-CGA = 0.724 vs. MPI = 0.765, p<0.0001) and one year of follow-up (areas under the ROC curves: FI-SOF = 0.694 vs. FI-CD = 0.729 vs. FI-CGA = 0.727 vs. MPI = 0.750, p<0.0001). The MPI showed a significant higher discriminatory power for predicting one-year mortality also in hospitalized older patients without functional limitations, without cognitive impairment, malnourished, with increased comorbidity, and with a high number of drugs.

**Conclusions:**

All frailty instruments were significantly associated with short- and long-term all-cause mortality, but MPI demonstrated a significant higher predictive power than other frailty instruments in hospitalized older patients.

## Introduction

In the last years, different conceptual definitions of frailty have been reported, i.e., phenotypic [1], accumulation of deficits [2], and multiple domain aggregate or multidimensional [3]. Mainly based on this last model, an integral conceptual working definition, taking into account essential components of existing conceptual definitions [4], [5], indicates frailty as a dynamic age-related condition of increased vulnerability characterized by declines across multiple physiologic systems and associated with an increased risk of negative outcomes, i.e., institutionalization and death [6]. Recently, frailty was demonstrated to be the most common condition leading to death in older people [7], suggesting that in clinical practice it is crucial to identify frailty in the older patient. Several instruments based on different conceptual approaches and validated in different settings and populations have been developed to detect frailty, and their predictive validity for mortality has also been established [8]. The different instruments, based on different conceptualization of frailty, however, capture different groups of older patients [9]. This is particularly problematic evaluating hospitalized older patients because prognostic information would be extremely useful in setting standard guidelines for care management, and follow-up after hospital discharge, and test their effectiveness [10].

In the present study, we compared four instruments, corresponding to the updated most widely accepted conceptual definitions of frailty [1]–[3], in the prediction of all-cause mortality of hospitalized older patients. For the phenotypic model, we used the frailty index derived from the Study of Osteoporotic Fractures (FI-SOF) [11], [12], which compared to the original frailty index as described by Fried and colleagues in the Cardiovascular Health Study (FI-CHS) is more easily implemented in a clinical setting [1], [13]. For the frailty index based on the cumulative deficits (FI-CD), we used the model described by Kulminski and colleagues [14]. For the multidimensional model, we used the frailty index based on a Comprehensive Geriatric Assessment (CGA) (FI-CGA) [15], [16] and the CGA-based Multidimensional Prognostic Index (MPI) [17]. CGA is defined as a multidimensional, interdisciplinary diagnostic process to determine the medical, psychological and functional capabilities of frail older people in order to develop a co-ordinated and integrated plan for treatment and long-term follow up [18], [19]. In particular, the FI-CGA explored ten domains (cognitive status, mood and motivation, communication, mobility, balance, bowel function, bladder function, functional status, nutrition, and social resources) and was validated on a randomized clinical trial [15] and the Canadian Study of Health and Aging, a large population-based study [16]. The MPI is a validated CGA-based algorithm that was developed on hospitalized older patients integrating data from eight domains including information on functional, cognitive, nutritional, comorbidities, drug use, risk of developing pressure sores, and co-habitation status [17].

Although validated on different settings (population-based studies, randomized clinical trial, or hospital-based settings), the criteria used to select the frailty instruments were their previous validation as prognostic tools for all-cause mortality in older people [11]–[17] and their feasibility in clinical practice [8]. The aim of the present study was to compare the accuracy of the four above-reported frailty instruments in predicting one-month and one-year all-cause mortality in hospitalized older patients.

## Materials and Methods

### Study population

The present was a prospective cohort study conducted according to the Declaration of Helsinki, the guidelines for Good Clinical Practice, and the Strengthening the Reporting of Observational Studies in Epidemiology (STROBE) guidelines (available at URL http://www.strobe-statement.org/). This study was approved by Institutional Review Boards (IRBs) of the twenty Italian Geriatric Wards involved and in particular by the IRBs of San Giovanni Rotondo (Foggia), Naples, Rome, Florence, and Turin. Written informed consent for research was obtained from each patient or from relatives or a legal guardian. Patients aged 65 years and older admitted from February 1 to March 31, 2008 due to acute disease or relapse of a chronic disease were screened for eligibility at 20 Italian geriatric units, homogeneously distributed on the Italian territory (eight from Northern Italy, four from Central Italy, and eight from Southern Italy). Inclusion criteria were: 1) age ≥65 years; 2) ability to provide an informed consent or availability of a proxy for informed consent; 3) complete data collection to carry out the four indexes during hospitalization. In particular, the information to calculate the four frailty indexes were collected in a single session during hospitalization, and the data to calculate activities of daily living (ADL) [20] and instrumental ADL (IADL) [21] were collected one time alone for both the FI-CGA and the MPI. The main and secondary diagnoses at discharge from the hospital, coded according to the Italian translation of the International Classification of Diseases, 9th revision, Clinical Modification (ICD-9-CM) (available at URL http://icd9cm.chrisendres.com/icd9cm/) were also recorded in all patients. Vital status up to April 1, 2009 was assessed by directly contacting the participants or consulting the Registry Offices of the cities where the patients were residents at the time of hospital admission.

### Frailty index derived from the Study of Osteoporotic Fractures (FI-SOF)

Some conceptual definitions of frailty are based on physical diminution in older people [1], [22]. The FI-CHS developed by Fried and colleagues is an operational definition of frailty in older subjects based on the presence of any three of the following five characteristics: shrinking, weakness, poor endurance, slowness, and low physical activity [1], so suggesting a phenotypic model of frailty. In the present study, for this phenotypic model, we selected the FI-SOF recently proposed by Ensrud and colleagues as a simpler index that might be more suitable for assessing frailty in a clinical practice setting [11], [12]. The FI-SOF was calculated on the basis of the following three items: a) unintentional weight loss, i.e., not due to diet or exercise, of more than 4.5 kg during the last year; b) inability to rise from a chair five times without the use of arms; c) low energy level as evaluated by the answer to the question “did you feel like you could not get going?”; those who reported that this feeling had occurred three days or more in the previous week were considered as demonstrating low energy level. Frailty status was defined as robust (0 components), prefrail (1 component), and frail (2 or 3 components) and expressed in three grades from grade 1 to grade 3 of frailty.

### Frailty index based on cumulative deficits (FI-CD)

Another conceptual approach to frailty suggests that an index based on health/well-being disorders (e.g., signs, symptoms, impairments, abnormal laboratory tests, diseases, etc.) accumulated by individuals during their life course can be considered as indicators of physiological frailty [2], [22]. The level of frailty can then be described by a composite measure of such disorders (called deficits), which can be determined for each individual as the fraction of deficits among a selected list of items that measure various aspects of health/well-being status. For this model of frailty, we selected the FI-CD [12] calculated considering a set of 32 deficits: difficulty with eating, dressing, walk around, getting in/out bed, getting bath, toileting, using telephone, going out, shopping, cooking, light house work, taking medicine, managing money, arthritis, Parkinson's disease, glaucoma, diabetes, stomach problems, history of heart attack, hypertension, history of stroke, flu, broken hip, broken bones, trouble with bladder/bowels, dementia, self-rated health, as well as problems with vision, hearing, ear, teeth, and feet. To calculate the index it is necessary to count the number of such deficits divided by the total number of all potential deficits considered for a given person. This index was expressed as a continuous variable.

### Frailty index based on a Comprehensive Geriatric Assessment (FI-CGA)

Some researchers have criticized the definitions of phenotypic frailty and a model based on cumulative deficits [5], [22], [23], suggesting that an integral conceptual approach is needed to identify and measure frailty in clinical practice, an approach in which the focus is not exclusively on the physical domains, but which also incorporates the evaluation of psychological and social domains, and is thus based on the integral functioning of the individual [4], [5]. For this multidimensional approach to frailty, we selected the FI-CGA [15], [16] calculated by counting the number of impairments identified in ten domains: 1) cognitive status; 2) mood and motivation, rated separately and then combined so that the highest level of specificity was scored for the domain; 3) communication, i.e. vision, hearing and speech; 4) mobility and 5) balance (each of the latter two scored at the highest level of independence also with the use of mobility or balance aids); 6) bowel function; 7) bladder function; 8) ADL [20], and IADL) [21], rated as no impairment = no problem, IADL impairment = mild problem, ADL impairment = major problem; 9) nutrition, and 10) social resources, scored as a problem if there was need for additional help. Problems in each domain were scored as 0 = no problem, 1 = minor problem, or 2 = major problem. The FI-CGA was expressed in three grades of frailty, i.e. FI-CGA 1 = mild, FI-CGA 2 = moderate and FI-CGA 3 = severe; the cut-off for mild, moderate and severe frailty were respectively 0–7, 7–13, and >13.

### Multidimensional Prognostic Index (MPI)

Finally, we also selected for the multidimensional and CGA-based model of frailty the MPI [17] calculated from information on eight domains including: 1) functional status assessed by the ADL [20] and 2) the IADL [21] scales; 3) cognitive status assessed by the Short Portable Mental Status Questionnaire (SPMSQ) [24]; 4) comorbidity as assessed by the Cumulative Illness Rating Scale (CIRS) [25]; 5) nutritional status according to the Mini Nutritional Assessment (MNA) [26]; 6) the risk of developing pressure sores assessed by the Exton Smith Scale (ESS) [27]; 7) the number of drugs taken by patients at admission and 8) co-habitation status, i.e. alone, in family or in institution. For each domain a tripartite hierarchy was used, i.e. 0 = no problems, 0.5 = minor problems, and 1 = major problems. The final MPI was expressed as three grades of risk of all-cause mortality: MPI-1 low risk (MPI value ≤0.33), MPI-2 moderate risk (MPI value between 0.34 and 0.66) and MPI-3 severe risk of all-cause mortality (MPI value >0.66). Table 1 summarized the methodological constructs of the four different frailty instruments.

**Table 1:** Summary of the methodological constructs of the four frailty indexes compared.

Frailty index	Evaluated parameters	Frailty determination	Conceptual approach
**FI-SOF (11, 12)**	3 items: 1) unintentional weight loss; 2) inability to rise from a chair five times without the use of arms; 3) low energy level	Robust: 0 component; prefrail: 1 component; frail 2 or 3 components. Only grading available	Phenotypic
**FI-CD (14)**	32 items: difficulty with eating, dressing, walk around, getting in/out bed, getting bath, toileting, using telephone, going out, shopping, cooking, light house work, taking medicine, managing money, arthritis, Parkinson's disease, glaucoma, diabetes, stomach problems, history of heart attack, hypertension, history of stroke, flu, broken hip, broken bones, trouble with bladder/bowels, dementia, self-rated health, as well as problems with vision, hearing, ear, teeth, and feet	The sum of the presence of deficits divided by the total number of all potential deficits. No grading available	Accumulation of deficits
**FI-CGA (13)**	10 domains: 1) cognitive status; 2) mood and motivation; 3) communication; 4) mobility; 5) balance; 6) bowel function; 7) bladder function; 8) basal ADL and IADL); 9) nutrition; 10) social resources	Problems in each domain were scored 0 (no problem), 1 (minor problem) and 2 (major problem). The sum determine the index. The cut-off for mild, moderate and severe frailty were respectively 0–7, 7–13 and >13	Multidimensional
**MPI (14)**	8 domains: 1) basal ADL; 2) IADL; 3) cognitive (SPMSQ); 4) comorbidity (CIRS); 5) nutrition (MNA); 6) risk of developing pressure sores (ESS); 7) the number of drugs taken by patients at admission; 8) co-habitation status	For each domain a tripartite hierarchy was used: 0 = no problems, 0.5 = minor problems and 1 = major problems. The sum was divided by the total number of the domains. The final MPI was expressed as three grades of risk of all-cause mortality: low risk (value ≤0.33), moderate risk (value between 0.34 and 0.66) and severe risk (value >0.66)	Multidimensional

FI-SOF: Frailty Index derived from the Study of Osteoporotic Fractures;

FI-CD: Frailty Index based on cumulative deficits;

FI-CGA: Frailty Index based on a Comprehensive Geriatric Assessment;

ADL: activities of daily living;

IADL: instrumental activities of daily living;

SPMSQ: Short Portable Mental Status Questionnaire;

CIRS: Cumulative Illness Rating Scale;

MNA: Mini Nutritional Assessment;

ESS: Exton-Smith Scale;

MPI: Multidimensional Prognostic Index.

### Statistical analysis

Patients' baseline characteristics were reported as mean ± standard deviation (SD) or frequencies and percentages for continuous and categorical variables, respectively. Baseline comparisons between men and women were assessed using generalized linear mixed-effects models accounting for clustering due to centre effect. Rank analysis were performed when skewness was present in continuous variables' distribution. Incidence rates (IR) for 100 person-month and 100 person-year over one-month and one-year of follow-up were also reported. Poisson regression models, accounting for clustering due to centre effect, were assessed to test differences in IR between men and women. Hazard ratios (HR) and 95% confidence intervals (95% CI) estimated from univariate proportional hazards regression models, accounting for clustering due to centre effect, were also shown. The discriminatory power, for one-month and one-year mortality, was assessed by estimating the area under the receiver operating characteristic (ROC) curves for crude and age- and sex-adjusted predictive models, accounting for clustering due to centre effect. Comparisons between the areas under the ROC curves were carried out using DeLong's test [28] both evaluating all patients and all possible subgroups defined by taking into account all clinical domains used to calculate the frailty indexes. Subgroups where some of these indexes showed a significant more or less discriminatory power than the others were reported. A *p* value<0.05 was considered for statistical significance. All analyses were performed using SAS Release 9.1 (SAS Institute, Cary, NC, USA).

## Results

### Characteristics of the study population

During the enrolment period, 2,322 consecutive patients were admitted to the 20 geriatric units and eligible for inclusion in the study. One hundred and eleven subjects were excluded because they were younger than 65 years, and 76 patients were excluded because data collection was not completed. Moreover, in 102 patients information on vital status after one year of follow-up was not available. Thus, the final analysis was performed in 2,033 patients, 874 men (43%) and 1159 women (57%). As shown in Table 2, the mean age was 79.8±7.8 years. As expected, women were significantly older (p<0.0001) and demonstrated an higher level of frailty than men. No significantly differences were observed between men and women for the main and secondary diagnoses at discharge. The overall mortality rates were 8.6% at one month and 24.9% at one year of follow-up, without significant differences between men and women.

**Table 2:** Baseline characteristics of hospitalized older patients according to gender.

	All	Men	Women	p-value**
Patients (n, %) †	2033 (100)	874 (43.0)	1159 (57.0)	----
Age (years) *	79.8±7.8	78.7±7.5	80.6±8.0	<0.0001
FI-SOF - Grade 1 (n,%) †	686 (33.7)	364 (41.6)	322 (27.8)	<0.0001
FI-SOF - Grade 2 (n,%) †	804 (39.5)	313 (35.8)	491 (42.4)	
FI-SOF - Grade 3 (n,%) †	543 (26.7)	197 (22.5)	346 (29.9)	
FI-CD *	10.3±6.2	9.5±6.0	10.9±6.3	<0.0001
FI-CGA - Grade 1 (n,%) †	1417 (69.7)	671 (76.8)	746 (64.4)	<0.0001
FI-CGA - Grade 2 (n,%) †	593 (29.2)	191 (21.9)	402 (34.7)	
FI-CGA - Grade 3 (n,%) †	23 (1.1)	12 (1.4)	11 (0.9)	
MPI - Grade 1 (n,%) †	851 (41.9)	429 (49.1)	422 (36.4)	<0.0001
MPI - Grade 2 (n,%) †	743 (36.5)	300 (34.3)	443 (38.2)	
MPI - Grade 3 (n,%) †	439 (21.6)	145 (16.6)	294 (25.4)	
ADL score *	3.8±2.5	4.2±2.4	3.5±2.5	<0.0001
IADL score *	3.7±3.1	3.9±3.0	3.6±3.2	0.1028
SPMSQ score *	2.8±3.0	2.4±3.0	3.1±3.1	<0.0001
Exton Smith score *	15.6±3.7	16.3±3.6	15.1±3.6	<0.0001
CIRS-CI score *	3.3±1.9	3.3±2.0	3.3±1.9	0.7495
MNA score *	20.5±5.8	21.4±5.7	19.9±5.8	<0.0001
Number of drugs *	4.5±2.7	4.4±2.8	4.6±2.7	0.1656
**Main Diagnoses**				
Diseases of the circulatory system (n,%)††	505 (24.8)	228 (26.1)	277 (23.9)	0.6415
Diseases of the respiratory system (n,%)	329 (16.2)	169 (19.3)	160 (13.8)	0.2336
Cerebrovascular disease (n,%)	298 (14.7)	136 (15.6)	162 (14)	0.8227
Disease of the digestive system (n,%)	209 (10.3)	83 (9.5)	126 (10.9)	0.9258
Disease of the nervous system (n,%)	208 (10.2)	78 (8.9)	130 (11.2)	0.7701
**Secondary diagnoses**				
Essential hypertension (n, %)	553 (27.2)	206 (23.6)	347 (29.9)	0.1324
Diabetes mellitus (n, %)	387 (19)	154 (17.6)	233 (20.1)	0.6311
Cardiac dysrhythmias (n, %)	382 (18.8)	153 (17.5)	229 (19.8)	0.6676
Chronic bronchitis (n, %)	335 (16.5)	182 (20.8)	153 (13.2)	0.0918
Chronic ischemic heart disease (n,%)	278 (13.7)	151 (17.3)	127 (10.9)	0.1791
Cerebral atherosclerosis (n,%)	265 (13)	111 (12.7)	154 (13.3)	0.9667
Hypertensive heart disease (n, %)	225 (11.1)	86 (9.8)	139 (12)	0.7707
Mortality - 1 month (events/pm, %ir)	165/1927 (8.6)	77/828 (9.3)	88/1099 (8.0)	0.7328
Mortality - 1 year (events/py, %ir)	430/1725 (24.9)	201/732 (27.4)	229/993 (23.0)	0.7617

*continuous variables;

† categorical variables; pm = person-month; py = person-years; ir = incidence rate.

**p-values obtained fitting generalized linear mixed-effects models, using variable rank values, accounting for clustering due to centre effect.

†† Excluding cerebrovascular disease.

FI-SOF: Frailty Index derived from the Study of Osteoporotic Fractures; FI-CD: Frailty Index based on cumulative deficits; FI-CGA: Frailty Index based on a Comprehensive Geriatric Assessment; ADL: activities of daily living; IADL: instrumental activities of daily living; SPMSQ: Short Portable Mental Status Questionnaire; CIRS: Cumulative Illness Rating Scale; MNA: Mini Nutritional Assessment;

ESS: Exton-Smith Scale; MPI: Multidimensional Prognostic Index.

### Comparison among different frailty instruments

**Figure 1:**
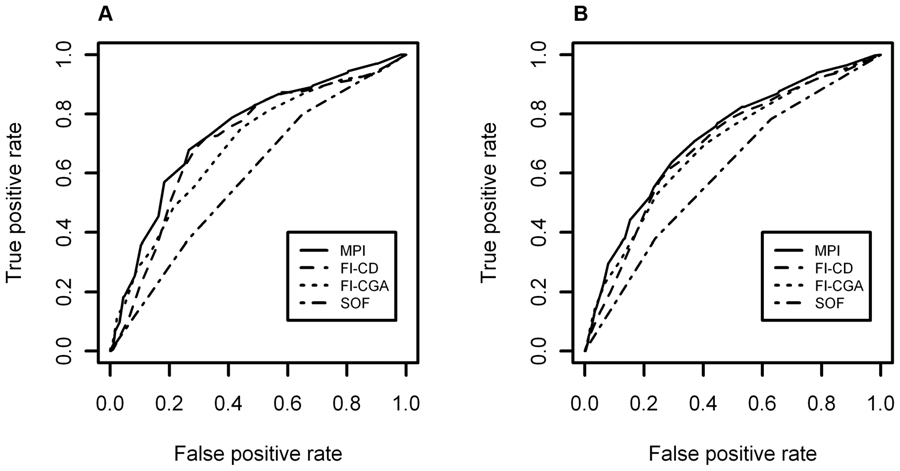
Comparisons among different frailty instruments on one-month and one-year all-cause mortality in hospitalized older patients. **Panel A** Receiver operating characteristic (ROC) curve comparisons among Multidimensional Prognostic Index (MPI), Frailty Index derived from the Study of Osteoporotic Fractures (FI-SOF), Frailty Index based on cumulative deficits (FI-CD), and Frailty Index based on a Comprehensive Geriatric Assessment (FI-CGA) scores on one-month all-cause mortality in hospitalized older patients. **Panel B** ROC curve comparisons among MPI, FI-SOF, FI-CD, and FI-CGA scores on one-year all-cause mortality in hospitalized older patients.

As shown in Table 3, all the frailty instruments were significantly associated with one-month and one-year all-cause mortality. Table 4 shows the areas under the ROC curves of the four frailty instruments investigated for one-month and one-year mortality from both crude and age- and sex-adjusted logistic regression models, accounting for clustering due to centre effect (Figure 1). The MPI demonstrated a significant higher discriminatory accuracy than FI-SOF, FI-CD, and FI-CGA after one month (areas under the ROC curves: SOF = 0.685 vs FI-CD = 0.738 vs FI-CGA = 0.724 vs MPI = 0.765, p<0.0001) and one year of follow-up (areas under the ROC curves: FI-SOF = 0.694 vs. FI-CD = 0.729 vs. FI-CGA = 0.727 vs. MPI = 0.750, p<0.0001). No differences in accuracy were observed between the FI-CD and the FI-CGA, while the FI-SOF demonstrated a lower accuracy than either FI-CD and FI-CGA both after one-month and one-year of follow-up (Table 4).

**Table 3:** Risk of one-month and one-year all-cause mortality according to the four frailty instruments in hospitalized older patients.

Follow-up	Frailty index	HR	95% CI	p-value**
	FI-SOF-1	1.00		
	FI-SOF-2	1.87	1.27–2.76	0.0016
	FI-SOF-3	2.42	1.16–5.04	0.0184
	FI-CD*	1.13	1.10–1.16	<0.0001
	FI-CGA-1	1.00		
**1-month follow-up**	FI-CGA-2	2.92	1.84–4.64	<0.0001
	FI-CGA-3	4.54	1.68–12.24	0.0028
	MPI-1	1.00		
	MPI-2	2.05	1.40–3.00	0.0002
	MPI-3	7.70	5.73–10.34	<0.0001
	FI-SOF-1	1.00		
	FI-SOF-2	1.67	1.29–2.17	<0.0001
	FI-SOF-3	2.45	1.44–4.18	<0.001
	FI-CD*	1.11	1.09–1.13	<0.0001
	FI-CGA-1	1.00		
**1-year follow-up**	FI-CGA-2	2.93	2.25–3.83	<0.0001
	FI-CGA-3	4.18	2.10–8.34	<0.0001
	MPI-1	1.00		
	MPI-2	2.00	1.64–2.45	<0.0001
	MPI-3	5.70	4.49–7.22	<0.0001

*continuous variables.

**p-values obtained fitting univariate proportional hazard regression models, accounting for clustering due to centre effect.

HR: hazard ratio; CI: confidence intervals; FI-SOF: Frailty Index derived from the Study of Osteoporotic Fractures; FI-CD: Frailty Index based on cumulative deficits; FI-CGA: Frailty Index based on a Comprehensive Geriatric Assessment; MPI: Multidimensional Prognostic Index.

**Table 4:** Comparison of the areas under the receiver operating characteristic (ROC) curves of the four frailty instruments compared.*

			Frailty index	AUC	SE	95% CI	Contrast p-value		
Follow-up	Crude/Adjusted Models						1 vs 2	1 vs 3	1 vs 4
			1.MPI	0.7486	0.0209	0.71–0.79			
		**Overall**	2. FI-SOF	0.5918	0.0208	0.55–0.63	<0.0001	<0.0001	<0.0001
			3.FI-CD	0.7094	0.0208	0.67–0.75			
			4.FI-CGA	0.6924	0.0218	0.65–0.73			
			1.MPI	0.7396	0.0332	0.67–0.80			
		**Male**	2. FI-SOF	0.6163	0.0313	0.55–0.67	0.0002	0.0017	0.0094
	**Unadjusted**		3.FI-CD	0.7013	0.0333	0.64–0.77			
			4.FI-CGA	0.6923	0.0340	0.62–0.76			
**1 month**			1.MPI	0.7636	0.0260	0.71–0.81			
		**Female**	2. FI-SOF	0.5749	0.0279	0.52–0.63	<0.0001	0.0051	<0.0001
			3.FI-CD	0.7216	0.0259	0.67–0.77			
			4.FI-CGA	0.6994	0.0282	0.64–0.76			
			1.MPI	0.7655	0.0203	0.72–0.80			
	**Age- and sex-**	**Overall**	2. FI-SOF	0.6854	0.0221	0.64–0.73	<0.0001	0.0005	<0.0001
	**adjusted**		3.FI-CD	0.7383	0.0204	0.69–0.78			
			4.FI-CGA	0.7240	0.0215	0.68–0.77			
			1.MPI	0.7231	0.0141	0.69–0.75			
		**Overall**	2. FI-SOF	0.6034	0.0142	0.57–0.63	<0.0001	<0.0001	<0.0001
			3.FI-CD	0.6934	0.0143	0.66–0.72			
			4.FI-CGA	0.6890	0.0145	0.66–0.72			
	**Unadjusted**		1.MPI	0.7164	0.0213	0.67–0.76			
**1 year**		**Male**	2. FI-SOF	0.6196	0.0210	0.58–0.66	<0.0001	0.0010	0.0048
			3.FI-CD	0.6836	0.0221	0.64–0.73			
			4.FI-CGA	0.6855	0.0219	0.64–0.73			
			1.MPI	0.7416	0.0184	0.70–0.78			
		**Female**	2. FI-SOF	0.5974	0.0193	0.56–0.63	<0.0001	0.0005	0.0003
			3.FI-CD	0.7081	0.0184	0.67–0.74			
			4.FI-CGA	0.7042	0.0189	0.67–0.74			
			1.MPI	0.7507	0.0135	0.72–0.78			
	**Age- and sex-**	**Overall**	2. FI-SOF	0.6948	0.0142	0.67–0.72	<0.0001	<0.0001	<0.0001
	**adjusted**		3.FI-CD	0.7297	0.0138	0.70–0.76			
			4.FI-CGA	0.7278	0.0139	0.70–0.75			

AUC: areas under curve; SE: standard error; CI: confidence interval; MPI: Multidimensional Prognostic Index; FI-SOF: Frailty Index derived from the Study of Osteoporotic Fractures; FI-CD: Frailty Index based on cumulative deficits; FI-CGA: Frailty Index based on a Comprehensive Geriatric Assessment.

*AUCs were assessed by crude and adjusted logistic regression models, accounting for clustering due to centre effect.

### Multidimensional domain analysis in different frailty instruments

Table 5 showed subgroups of hospitalized older patients where the different frailty instruments demonstrated a significant different predictive discriminatory power for one-year all-cause mortality according to the multidimensional domain analysis. The MPI showed a significant higher discriminatory power for prediction of one-year mortality than the other indexes in patients without functional limitations (ADL = 5–6 and IADL = 6–8) or cognitive impairment (SPMSQ = 0–3). The MPI had also a significant higher prognostic accuracy than other frailty instruments in hospitalized patients with malnutrition (MNA<17), higher level of comorbidity (CIRS≥3), and in those who were taking a higher number of drugs (≥7) (Table 5).

**Table 5:** Subgroups of hospitalized older patients where different frailty indexes showed a significant different predictive discriminatory power for one-year all-cause mortality.*

	Frailty index	AUC	SE	95%	Contrast p-value		
					1 vs 2	1 vs 3	1 vs 4
	1 – MPI	0.7086	0.0199	0.67–0.75			
**ADL 6-5**	2 – FI-SOF	0.6553	0.0210	0.61–0.70	0.0007	0.0004	0.0015
**(N = 714)**	3 - FI-CD	0.6581	0.0209	0.62–0.70			
	4 - FI-CGA	0.6667	0.0208	0.63–0.71			
	1 – MPI	0.7277	0.0169	0.69–0.76			
**IADL 8-6**	2 – FI-SOF	0.6569	0.0181	0.62–0.69	<0.0001	<0.0001	0.0003
**(N = 1038)**	3 - FI-CD	0.6820	0.0176	0.65–0.72			
	4 - FI-CGA	0.6897	0.0178	0.65–0.72			
	1 – MPI	0.7490	0.0170	0.71–0.78			
**SPMSQ 0-3**	2 – FI-SOF	0.7012	0.0178	0.67–0.74	0.0001	0.0012	0.0007
**(N = 1503)**	3 - FI-CD	0.7279	0.0175	0.69–0.76			
	4 - FI-CGA	0.7237	0.0176	0.69–0.76			
	1 – MPI	0.7492	0.0163	0.72–0.78			
**CIRS≥3**	2 – FI-SOF	0.6814	0.0173	0.65–0.71	<0.0001	<0.0001	0.0002
**(N = 1257)**	3 - FI-CD	0.7202	0.0167	0.69–0.75			
	4 - FI-CGA	0.7197	0.0170	0.69–0.75			
	1 – MPI	0.7374	0.0218	0.69–0.78			
**MNA<17**	2 – FI-SOF	0.6833	0.0234	0.64–0.73	0.0007	0.0651	0.0669
**(N = 534)**	3 - FI-CD	0.7164	0.0221	0.67–0.76			
	4 - FI-CGA	0.7146	0.0224	0.67–0.76			
	1 – MPI	0.7069	0.0306	0.65–0.77			
**Number of drugs ≥7**	2 – FI-SOF	0.6473	0.0321	0.58–0.71	0.0197	0.3952	0.0073
**(N = 463)**	3 - FI-CD	0.6972	0.0313	0.63–0.76			
	4 - FI-CGA	0.6712	0.0317	0.61–0.73			

AUC: areas under curve; SE: standard error; CI: confidence interval; ADL: activities of daily living; MPI: Multidimensional Prognostic Index; FI-SOF: Frailty Index derived from the Study of Osteoporotic Fractures; FI-CD: Frailty Index based on cumulative deficits; FI-CGA: Frailty Index based on a Comprehensive Geriatric Assessment; IADL: instrumental activities of daily living; SPMSQ: Short Portable Mental Status Questionnaire; CIRS: Cumulative Illness Rating Scale;

MNA: Mini Nutritional Assessment.

*AUCs were assessed by crude and adjusted logistic regression models, accounting for clustering due to centre effect.

## Discussion

In the present study, we demonstrated that the MPI was more effective than the other frailty instruments in predicting short- and long-term all-cause mortality risk in hospitalized older patients admitted to geriatric units. Furthermore, the MPI showed a significant higher discriminatory power for prediction of one-year all-cause mortality than the other frailty indexes in some subgroups of hospitalized older patients, i.e., those without functional limitations, those without cognitive impairment, those malnourished, with higher level of comorbidity, and those who were taking an high number of drugs.

Cumulative clinical evidence suggested that the MPI has demonstrated its validity and accuracy in predicting all-causes mortality in several previous studies carried out in hospitalized older patients with acute diseases or relapse of a chronic disease such as gastrointestinal bleeding [29], pneumonia [30], heart failure [31], chronic kidney disease [32], liver cirrhosis [33], and dementia [34]. In the present cohort, women demonstrated a significantly higher level of frailty than men, as detected by all the four prognostic tools investigated. On the other hand, male gender was associated with an increased one-year all-cause mortality, although not significantly, compared to female gender. These findings confirmed our previous data [17], [35] and are in agreement also with other studies from American [14], Canadian [36], and Chinese older populations [37], which reported that the gender difference in life expectancy was not entirely due to differences in impairment, because men with the same chronological age and biological impairments (e.g., frailty index) had a higher risk of death compared with that of women.

The present findings showed further evidence that a multiple-domain based instrument of frailty, such as the MPI, may be more effective in predicting short- and long-term all-cause mortality in hospitalized older patients than other instruments based on different frailty models, i.e., physical diminution in older persons (FI-SOF) [11], [12] or a deficit accumulation approach (FI-AD) [14]. The present findings also showed that the MPI had higher predictive discriminatory power than a CGA-based tool (FI-CGA) [15], [16]. However, this last frailty index was calculated as a count of the impairments identified with the CGA [13], while the MPI was mainly based on standardized assessment instruments widely employed in geriatric practice, that comprehensively explored different domains (ADL, IADL, SPMSQ, CIRS, MNA, and ESS), so partly explaining the differences of these two tools in predicting all-cause mortality in this population of hospitalized older patients. A captivating aspect of the MPI is that it comprises not only a grading system but a continuous one. This permits to perform a more fine analysis in settings where it may be necessary to verify not only the macroscopic change in mortality but also different risks at different times. This is possible because the MPI integrates different domains that could change over time reflecting the current health status in older people. The integrated and multidimensional conceptualization of frailty clearly presents advantages over the other constructs in the recognition of level of frailty in hospitalized older patients considering short- and long-term all-cause mortality as the primary outcome. A holistic and dynamic model of frailty may be central in the management and care of these patients [5]. In fact, in the present study, the worst performance was achieved by the FI-SOF [1], [11]–[13], that included only physical components and not also psychological, cognitive, and social factors.

A very recent systematic review evaluated clinimetric properties and searched for the best available frailty instrument that can be used as an evaluative outcome measure in clinical practice and that may be useful in observational and experimental studies [8]. Based on recent studies [3], [5], [15], a list of eight frailty risk factors that are mentioned to be of great importance to the concept of frailty were identified [8], including in the physical dimension: nutritional status, physical activity, mobility, strength and energy, in the psychological dimension: cognition and mood, and in the social dimension: lack of social contacts and social support. On this basis, at least twenty frailty instruments have been described [8], and all these frailty instruments are multidimensional in nature, and mostly based on a standardized CGA [8]. However, the overall results of the assessment by using these frailty instruments, suggested that they are mainly developed and validated as risk assessment tools, and not as possible outcome measures [8]. At the best of our knowledge, this was the first multicentre study that compared four frailty indexes based on three conceptual approaches in a population of older hospitalized patients. Some studies made a comparison among these frailty instruments [9], [11]–[13], [38]–[42], ^but^ in population-based settings [9], [11]–[13], [38], [40] or in home-care settings [39], [42], considering hospitalized older patients only for particular diagnostic categories (e.g., coronary artery disease) [41]. In particular, a large population-based study made a comparison among a functional domain model, a cumulative deficit model, and a phenotypic model suggesting that different theoretical constructs of frailty may capture different groups of older adults, with some overlap [9]. Furthermore, the comparison of two phenotypic frailty indexes suggested that the simple FI-SOF based on three components and the more complex FI-CHS [1], [13] performed similarly in predicting falls, disability, fractures, and mortality on both osteoporotic women [11] and men in the Osteoporotic Fractures in Men Study [12]. In an hospital-based setting, a study compared two phenotypic frailty indexes but only in older patients with coronary artery disease [41]. Therefore, it is becoming apparent a central role of the evaluation of frailty indexes in hospital-based settings with high risk of mortality and in which other than the acute phase care is important the choice of the appropriate long-term treatment and management after the hospital discharge.

This study has several strengths, including its large and multicentre sample, its prospective design, comprehensive set of measurements, and completeness of follow-up. Notwithstanding these interesting findings, we must acknowledge that the present study had some limitations. In fact, since the study population included only patients admitted to geriatric units, a generalization of the present findings also in other settings must be validated. Furthermore, the other tested frailty instruments were originally validated on different populations and settings [11], [12], [14]–[16], and not in an hospital-based setting. However, we selected these tools for the lack of instruments widely diffused and validated in this setting, also considering the possibility of an application in the clinical practice. On the other hand, also other studies conducted in home-care [39], [42] or hospital-based settings [41] made a comparison among frailty instrument previously validated in other settings. Finally, at present, differently from the other frailty instruments selected for this study [11], [12], [14]–[16], we have no data on the usefulness of the MPI in identifying older adults at high risk of adverse outcomes traditionally linked to frailty such as falls or institutionalization, although this instrument was based on standardized assessment instruments widely employed in the CGA. Indeed, the MPI is a valid and accurate predictor of all-causes mortality in hospitalized older patients with acute diseases or relapse of a chronic diseases [29]–[35]. In the present study, we only evaluated the effectiveness of different tools in predicting short- and long-term all-cause mortality risk in hospitalized older patients, avoiding to study other outcomes traditionally linked to frailty.

### Conclusions

The present study suggests that a multidimensional and integral conceptual model of frailty, taking into account not exclusively physical problems in older people, but also psychological, cognitive, and social components of frailty, and thus based on the integral functioning of the individual, may have higher predictive power for adverse outcomes and particularly all-cause mortality in clinical settings. In the next future, it will be of interest to consider also the causes of hospital admission and the diagnoses at discharge, and to separate the causes of mortality when evaluating accuracy and predictivity of different frailty instruments in different settings.

## Supporting Information

Appendix S1 **Fondazione Italiana per la Ricerca sull'Invecchiamento and Gerontology and Geriatrics Italian Society (FIRI-SIGG) Study Group Investigators and Institutional Review Boards that approved the study protocol.**
(DOC)
