# Supplementary material for: Comparing the Prognostic Accuracy for All-Cause Mortality of Frailty Instruments: A Multicentre 1-Year Follow-Up in Hospitalized Older Patients
Source: PLoS One. 2012 Jan 11;7(1):e29090. doi: 10.1371/journal.pone.0029090 (PMC3256139; doi:10.1371/journal.pone.0029090)
Supplement: Appendix S1 — Fondazione Italiana per la Ricerca sull'Invecchiamento and Gerontology and Geriatrics Italian Society (FIRI-SIGG) Study Group Investigators and Institutional Review Boards that approved the study protocol. (DOC) [file pone.0029090.s001.doc]

**Appendix S1.**

**FIRI-SIGG Study Group Investigators and Institutional Review Boards that approved the study protocol**

1. S.O.C. Geriatric Unit, “S. Marta S. Venera” Hospital - Acireale (CT), Italy: Santo Branca MD, Tiziana De Gregorio MD, Mauro Manmano MD, Giacomo Spallina MD;
2. S.O.C. Geriatric Medicine ASO - Alessandria, Italy: Enzo Laguzzi MD, Giorgio Estienne MD, Aurelio Massone MD, Maria Moscato MD, Carla Ravera MD;
3. Geriatric Unit INRCA-IRCCS Ancona, Italy: Letizia Ferrara MD, Giuseppe De Tommaso MD, David Serenella MD;
4. Geriatric Unit, **Poliambulanza Hospital - Brescia, Italy**: Renzo Rozzini MD, Piera Barbisoni MD, Intissar Sleiman MD;
5. Geriatric Unit “A.Perrino” Hospital - Brindisi, Italy: Vito Carrieri MD, Carmine Devicienti MD, Roberto Cristofalo MD, Giovanni Argentieri MD;
6. Geriatric Unit, S. Orsola-Malpighi Hospital - Bologna, Italy: Afro Salsi MD, Lorenzo Bellotti MD, Roberto Bernardi MD, Francesco Nicolino MD;
7. Geriatric Unit, SS Trinità Hospital - Cagliari, Italy: Paolo F. Putzu MD, Giuseppina Caddeo MD, Francesca Coghe MD, Daniela Riccio MD;
8. Geriatric Unit, I.N.R.C.A- Cosenza, Italy: Bruno Mazzei MD, Andrea Corsonello MD;
9. Unit of Gerontology and Geriatrics, Department of Critical Care Medicine and Surgery, University of Florence and Azienda Ospedaliero-Universitaria Careggi, Florence, Italy: Mauro Di Bari, MD, PhD, Francesca Caldi, MD, Eugenia Lopilato, MD, Niccolò Marchionni, MD, Irene Tassinari, MD;
10. Geriatric Unit, Madonna delle Grazie Hospital - Matera, Italy: Salvatore Tardi MD, Eugenia Piermichela Mascolo MD;
11. Geriatric Division, Department of Clinical Medicine, Cardiovascular and immunolgical Sciences, Cardiovascular University of Naples “Federico II”, Italy: Pasquale Abete MD, PhD, Franco Rengo MD, Immacolata Simione MD;
12. Geriatric Unit, “P.F. Calvi” Hospital – Noale, Italy: Romano Centomo MD, Alberto Cester MD, Moreno Scevola MD;
13. Geriatric Unit, Hospital of Parma - Parma, Italy: Pia Lunardelli MD, Antonio Giordano MD, Emilio Martini MD, Anna Nardelli MD, Sandra Visioli MD;
14. Geriatric Institute, Policlinico Santa Maria della Misericordia, University of Perugia – Perugia, Italy: Antonio Cherubini MD, PhD, Giusy Dell’Aquila MD, Beatrice Gasperini MD, Umberto Senin MD;
15. Geriatric Unit,”Misericordia e Dolce” Hospital - Prato: Antonio Mitidieri Costanza MD, Antonio Bavazzano MD, Lucia Gambardella MD, Novella Malin MD;
16. Geriatric Department, Catholic University, Rome, Italy: Roberto Bernabei MD, Carola D’Arco MD, Giovanni Gambassi MD, Federica Mammarella MD;
17. Department of Medical Sciences - Geriatric Unit, IRCCS “Casa sollievo della Sofferenza”- San Giovanni Rotondo: Alberto Pilotto MD, Leandro Cascavilla MD, Francesco Paris MD, Carlo Scarcelli MD;
18. Geriatric Unit, De Lellis Hospital – Schio (VI), Italy: Carla Grasselli MD, Paolo Brunello, Cristina Cortiana, Daniela Pavin MD;
19. Geriatric Unit , Birago di Vische-ASL TO2 Hospital - Torino, Italy: Sergio Cabodi MD, Rosaria Carlucci MD, Davide Grassone MD;
20. Geriatric Unit, Cattinara Hospital, University of Trieste - Trieste, Italy: Gabriele Toigo MD, Paolo De Colle MD, Luca Lattuada MD, Alessandro Tulliani MD.
